# Integrated safety of levodopa‐carbidopa intestinal gel from prospective clinical trials

**DOI:** 10.1002/mds.26485

**Published:** 2015-12-23

**Authors:** Anthony E. Lang, Ramon L. Rodriguez, James T. Boyd, Sylvain Chouinard, Cindy Zadikoff, Alberto J. Espay, John T. Slevin, Hubert H. Fernandez, Mark F. Lew, David A. Stein, Per Odin, Victor S.C. Fung, Fabian Klostermann, Alfonso Fasano, Peter V. Draganov, Nathan Schmulewitz, Weining Z. Robieson, Susan Eaton, Krai Chatamra, Janet A. Benesh, Jordan Dubow

**Affiliations:** ^1^ Morton and Gloria Shulman Movement Disorders Clinic and the Edmond J. Safra Program in Parkinson's Disease, Toronto Western Hospital and Division of Neurology, UHN, Division of NeurologyUniversity of TorontoTorontoOntarioCanada; ^2^ University of Florida College of MedicineGainesvilleFloridaUSA; ^3^ University of Vermont College of MedicineBurlingtonVermontUSA; ^4^ University of MontrealMontrealQuebecCanada; ^5^ Feinberg School of MedicineNorthwestern UniversityChicagoIllinoisUSA; ^6^ University of Cincinnati Academic Health CenterCincinnatiOhioUSA; ^7^ University of Kentucky Medical CenterLexingtonKentuckyUSA; ^8^ Center for Neurological Restoration, Cleveland ClinicClevelandOhioUSA; ^9^ Keck/University of Southern California School of MedicineLos AngelesCaliforniaUSA; ^10^ QuintilesSan DiegoCaliforniaUSA; ^11^ Klinikim‐BremerhavenGermany and Skane University HospitalLundSweden; ^12^ Westmead Hospital and Sydney Medical SchoolSydneyAustralia; ^13^ Charité‐University Medicine BerlinBerlinGermany; ^14^ AbbVie IncNorth ChicagoIllinoisUSA

**Keywords:** Levodopa‐carbidopa intestinal gel, infusion, safety, percutaneous endoscopic gastrojejunostomy, Parkinson's disease

## Abstract

**Background:**

Continuous administration of levodopa‐carbidopa intestinal gel (carbidopa‐levodopa enteral suspension) through a percutaneous endoscopic gastrojejunostomy is a treatment option for advanced Parkinson disease (PD) patients with motor fluctuations resistant to standard oral medications. Safety data from 4 prospective studies were integrated to assess the safety of this therapy.

**Methods:**

Safety data from 4 studies were summarized using 2 overlapping data sets, permitting the separation of procedure/device–associated (n = 395) from non‐procedure/device adverse events (n = 412).

**Results:**

At the data cutoff, median exposure to levodopa‐carbidopa intestinal gel was 911 days (range, 1‐1980 days) with 963 total patient‐years of exposure. Procedure/device adverse events occurred in 300 patients (76%), and serious adverse events occurred in 68 (17%); most frequently reported procedure/device adverse events and serious adverse events were complications of device insertion (41% and 8%, respectively) and abdominal pain (36% and 4%, respectively). Non‐procedure/device adverse events occurred in 92% (379), with most frequently reported being insomnia (23%) and falls (23%); 42% (171) had non‐procedure/device serious adverse events, with most frequently reported being pneumonia (5%) and PD symptoms (2%). Adverse events led to discontinuation in 17% (72), most frequently because of complication of device insertion (2.4%). There were 34 treatment‐emergent deaths (8.3%) in the overlapping data sets, 2 of which (0.5%) were considered “possibly related” to the treatment system.

**Conclusion:**

In the largest collection of levodopa‐carbidopa intestinal gel safety data from prospective clinical studies, procedure/device events were frequently reported and occasionally life threatening. Most non‐procedure/device events were typical for levodopa treatment and an elderly population. These factors combined with high treatment efficacy led to a relatively low discontinuation rate in advanced PD patients. © 2015 International Parkinson and Movement Disorder Society

Although oral levodopa is the most common and effective treatment for Parkinson's disease (PD), it has been reported that disabling motor complications occur in nearly 90% of patients after 9 years of treatment.^1^ Advanced PD patients cycle between periods of poor mobility (“off” time), and good mobility (“on” time) with or without dyskinesias.^2^ The pathophysiological reason for this on‐off phenomenon is poorly understood; however, critical factors include the pulsatile availability of dopamine and fluctuating plasma concentrations of levodopa^3^ because of its short half‐life and erratic gastric emptying.^4^ “Off” and “on” times with troublesome dyskinesia reduce patients' quality of life and put them at higher risk of comorbid complications.^5^, ^6^

The levodopa‐carbidopa intestinal gel (LCIG) system continuously infuses levodopa and carbidopa in a carboxymethylcellulose aqueous gel to the proximal small intestine with the intent of minimizing the variability in plasma levodopa levels caused by unpredictable gastric emptying. LCIG is administered by a portable infusion pump through a percutaneous endoscopic gastrostomy with jejunal extension (PEG‐J). Initial pharmacokinetic studies comparing LCIG with oral formulations of levodopa demonstrated that LCIG provided a less variable plasma level of levodopa.^7^, ^8^ In a multinational, randomized, double‐blind clinical study in advanced PD patients, LCIG resulted in a significant improvement in “off” time, “on” time without troublesome dyskinesia, and measures of quality of life and activities of daily living compared with immediate‐release oral levodopa‐carbidopa (IR‐LC).^9^

LCIG has been commercially available in Europe since 2004, and although the safety of the procedure may be appreciated outside clinical trials, the available long‐term, prospective safety data for LCIG are limited. Given that treatment with LCIG involves both a drug and a device that requires an endoscopic procedure, safety is particularly important when assessing its risk/benefit profile. To evaluate the safety specific to the procedure/device separate from the levodopa‐exposure or underlying disease, data from 4 phase 3 studies were integrated and analyzed as 2 separate data sets. This report summarizes the largest, longest‐term data set on LCIG safety presented to date including more than 400 patients with an average exposure of more than 2 years.

## Methods

### LCIG Phase 3 Studies

There were 4 prospective, multicenter phase 3 LCIG (designated in the United States as carbidopa‐levodopa enteral suspension [CLES]) studies (NCT00357994/NCT00660387, NCT00335153, NCT00660673, NCT00360568), 3 of which have been previously reported 9, 10, 11 and a fourth that is ongoing. The study protocols were approved by the institutional review board/ethics committee at all 90 centers in 16 countries, and all patients provided written informed consent.

### Studies and Patients Included in Analysis

The enrolled patients were adults (≥30 years old) with advanced PD consistent with UK Brain Bank criteria.^9^ Patients were levodopa‐responsive with PD complicated by severe motor fluctuations uncontrolled by optimized standard therapies.^9^ In all studies, LCIG was administered to patients during 16 waking hours. Ninety‐six percent of the 412 patients received concomitant anti‐PD medication for any period of time during LCIG treatment, and the most frequent were oral levodopa (83%) and amantadine (28%).

**Figure 1:**
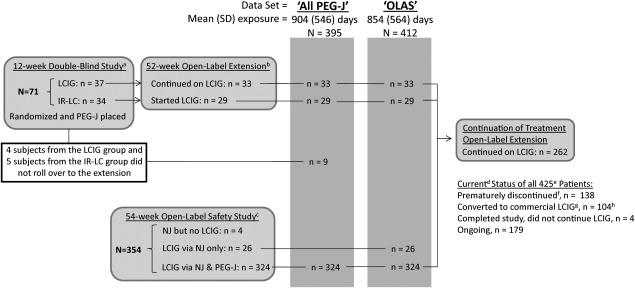
Flowchart of patient populations in the study data sets. All PEG‐J, data set of patients who had PEG‐J placement; OLAS, open‐label LCIG analysis data set; PEG‐J, percutaneous endoscopic gastrojejunostomy; LCIG, levodopa‐carbidopa intestinal gel; IR‐LC, immediate‐release oral levodopa‐carbidopa; NJ, nasojejunal tube. a. Olanow et al. Lancet Neurol. 2014; 13(2):141‐9.^9^ b. Slevin et al. J Parkinson's Dis. 2015; 5(1):165‐74.^11^ c. Fernandez et al. Mov Disord. 2015; 30(4):500‐9.^10^ d. As of March 31, 2014. e. 71 patients from the double‐blind study and 354 from the open‐label safety study. f. Discontinued for any reason, including AE, lack of efficacy, withdrew consent, administrative, or protocol violation. g. An estimate based on patients who completed a study, did not roll over into an extension study, did not have their PEG‐J removed and lived in a country where LCIG was commercially available. Patients who lived in a country where LCIG was commercially available were not allowed to participate in the continuing of treatment extension. h. 4 converted to commercial LCIG after completing the double‐blind study, and 92 after participating in an open‐label study.

The 4 studies from which data were integrated in this analysis were (1) a 12‐week double‐blind, double‐dummy study,^9^ (2) a 52‐week open‐label extension of the double‐blind, double‐dummy study,^11^ (3) a 54‐week open‐label safety study,^10^ and (4) an ongoing open‐label extension study providing continued access to treatment (Fig. 1). Briefly, 66 of the 71 enrolled patients (93%) completed the double‐blind, double‐dummy study in which all randomized patients had a PEG‐J placed before receiving either LCIG infusion and oral placebo capsules or placebo gel infusion and encapsulated IR‐LC tablets; 62 patients elected to participate in the subsequent 52‐week open‐label extension, in which all participants received LCIG via PEG‐J,^11^ after which 48 of these continued in the ongoing extension study (Fig. 1). In a separate open‐label safety study, LCIG was initially titrated via nasojejunal (NJ) tube in 350 patients; 324 of these (92%) proceeded to receive LCIG via PEG‐J. Of the 283 patients (87% of patients with a PEG‐J) who completed the open‐label safety study, 214 enrolled in the ongoing extension study (Fig. 1).^10^ As of March 31, 2014, 179 patients were continuing LCIG in the ongoing extension study (Fig. 1).

### PEG‐J Procedure

The PEG‐J placement procedure was performed by a qualified gastroenterologist, surgeon or interventional radiologist as previously described.^12^ Gastrointestinal proceduralists received standardized instructions and training prior to performing the PEG‐J procedure.

### Safety Assessments

All adverse events (AEs) that occurred during each study, regardless of their perceived relationship to the treatment system (drug or device), were recorded, coded according to Medical Dictionary for Regulatory Activities (MedDRA) version 14.0^13^ and tabulated by MedDRA Preferred Terms (PTs). AEs were monitored from screening and could be coded to more than 1 PT descriptive of the event. All AEs were treatment emergent, defined as those that started or worsened on or after day 1 of exposure to the treatment system and within 30 days of the end of treatment. Study investigators rated each AE as serious^14^ (events that are life threatening, requiring/prolonging hospitalization beyond the required study procedures, or result in death, significant disability, or birth defect) or nonserious in nature and noted whether they considered the event to be “unrelated,” “unlikely,” “possibly,” or “probably” related to the treatment system. Complications related to the product quality of the infusion device (eg, pump, tubing) were recorded by the investigator as product quality device complaints, and although they may have been associated with an AE, device complaints were not themselves AEs.

AEs were categorized as either procedure/device AEs or non‐procedure/device AEs, but not both. Procedure/device‐associated AEs (procedure/device AEs) were identified using a MedDRA PT search strategy of terms potentially related to the procedure or use of the device, as determined by a medical review board of gastrointestinal specialists. Non‐procedure/device‐associated AEs (non‐procedure/device AEs) included all other AEs not associated with the procedure/device. Procedure/device AEs and non‐procedure/device AEs were analyzed in 2 separate but overlapping data sets (see below). Impulse control disorders (ICDs) were monitored with the Minnesota Impulsive Disorders Interview (MIDI) Assessment, and AEs related to ICDs were identified using a MedDRA PT search strategy composed of PTs associated with “abuse liability.” AEs related to neuropathy were assessed by a MedDRA PT search strategy defined by the polyneuropathy Standardized MedDRA Query.^15^

Hematologic and metabolic laboratory values, including folate, homocysteine, methylmalonic acid, and vitamins B_6_ and B_12_, were assessed every 3 to 6 months. Study investigators were not required to conduct nerve conduction studies. A gastrointestinal adjudication committee reviewed procedure/device‐associated safety data as previously reported.^16^

### Data Sets

Because of the differences in study design and discontinuations, not all patients had a PEG‐J placed or were exposed to LCIG. Consequently, 2 patient data sets were used to more acutely examine the safety of this drug/device system. The “All PEG‐J” data set (n = 395), used to analyze the safety of the gastrointestinal surgical procedure and complications associated with the device, consisted of patients who had the PEG‐J placed in any of the phase 3 studies, regardless of their exposure to LCIG (Fig. 1). The Open‐label Analysis Set (OLAS, n = 412), used to analyze the safety of LCIG, consisted of all patients who received open‐label LCIG treatment during one of the studies, whether infused via NJ or PEG‐J (Fig. 1). These data sets overlap except for 9 patients from the double‐blind, double‐dummy study who did not enroll in the open‐label extension (included in the All PEG‐J data set but not OLAS) and 26 patients from the open‐label safety study who only received LCIG via an NJ and did not go on to receive a PEG‐J (included in the OLAS but not the All PEG‐J data set); see Figure 1.

### Statistical Analyses

The safety databases with a cutoff date of March 31, 2014, from each individual study were integrated. This initial safety evaluation focused on summarizing the seriousness and temporal distribution of AEs associated or not associated with the procedure/device as well as discontinuations, deaths, device complaints, and tube replacements.

Prespecified subgroup analyses of the safety data with a cutoff date of May 31, 2013, were conducted for intrinsic (age [<65 and ≥65 years], race, sex, baseline body mass index [BMI], and duration of PD [<10 and ≥10 years]) and extrinsic factors (dopamine agonist use and region) and evaluated for 4 periods of AE occurrence: any time, titration period (onset during first 28 days), maintenance period (onset on or after day 29), and persistent (onset during titration and extended to maintenance period with a duration ≥ 7 days).

## Results

As summarized in Table 1, patients' baseline characteristics were similar between the overlapping data sets. The median (range) exposure to LCIG was 911 days (1‐1980 days) (OLAS; Supplemental Table 1). There were 336 patients (82%) treated with LCIG for at least 1 year and 233 (57%) for at least 2 years, with the total patient‐years of exposure exceeding 950 years (OLAS; Supplemental Table 1). Of the participants in the open‐label studies (n = 412), more than half (262, 64%) had rolled over into the ongoing extension study, with 179 (43%) still participating and 104 (25%) who likely converted to commercial LCIG (Fig. 1).

**Table 1:** Patients' baseline characteristics

Characteristic	All PEG‐J n = 395^a^	OLAS n = 412^b^
Age in years, mean ± SD	64.3 ± 8.8	64.1 ± 8.9
<65 years, n (%)	192 (49)	201 (49)
≥65 years, n (%)	203 (51)	211 (51)
Sex (male), n (%)	231 (59)	243 (59)
Race, n (%) White	367 (93)	381 (93)
Asian	24 (6.1)	26 (6.3)
Other	4 (1.0)	5 (1.2)
Mini‐Mental State Exam, mean ± SD	28.6 ± 1.6	28.5 ± 1.7
Duration of PD in years, mean ± SD	12.2 ± 5.5	12.3 ± 5.5
“Off” time (h/d), mean ± SD^c^	NC	6.7 ± 2.3
“On” time without troublesome dyskinesia (h/d), mean ± SD^c^	NC	7.8 ± 2.4
“On” time with troublesome dyskinesia (h/d), mean ± SD^c^	NC	1.5 ± 2.0
Daily oral levodopa dose (mg), mean ± SD^d^	NC	1080.7 ± 565.3

All PEG‐J, data set of patients who had PEG‐J placement; OLAS, open‐label LCIG analysis data set; h/d, hours per day; NC, not calculated.

a Includes 9 patients not in the OLAS group.

b Includes 26 patients not in the All PEG‐J group.

c n = 406 for diary measures.

d n = 410 for levodopa dose.

Overall, AEs (procedure/device *and* non‐procedure/device) were reported in 387 subjects (94%; n = 412), and SAEs (procedure/device *and* non‐procedure/device), were reported in 194 subjects (47%; n = 412). Procedure/device AEs and SAEs were analyzed separately from non‐procedure/device events. There were no clinically meaningful trends among demographic subgroups in the All PEG‐J (Supplemental Table 2) or OLAS (Supplemental Table 3) safety analyses.

### Procedure/Device‐Associated LCIG Safety (All PEG‐J Data Set, n = 395)

**Figure 2:**
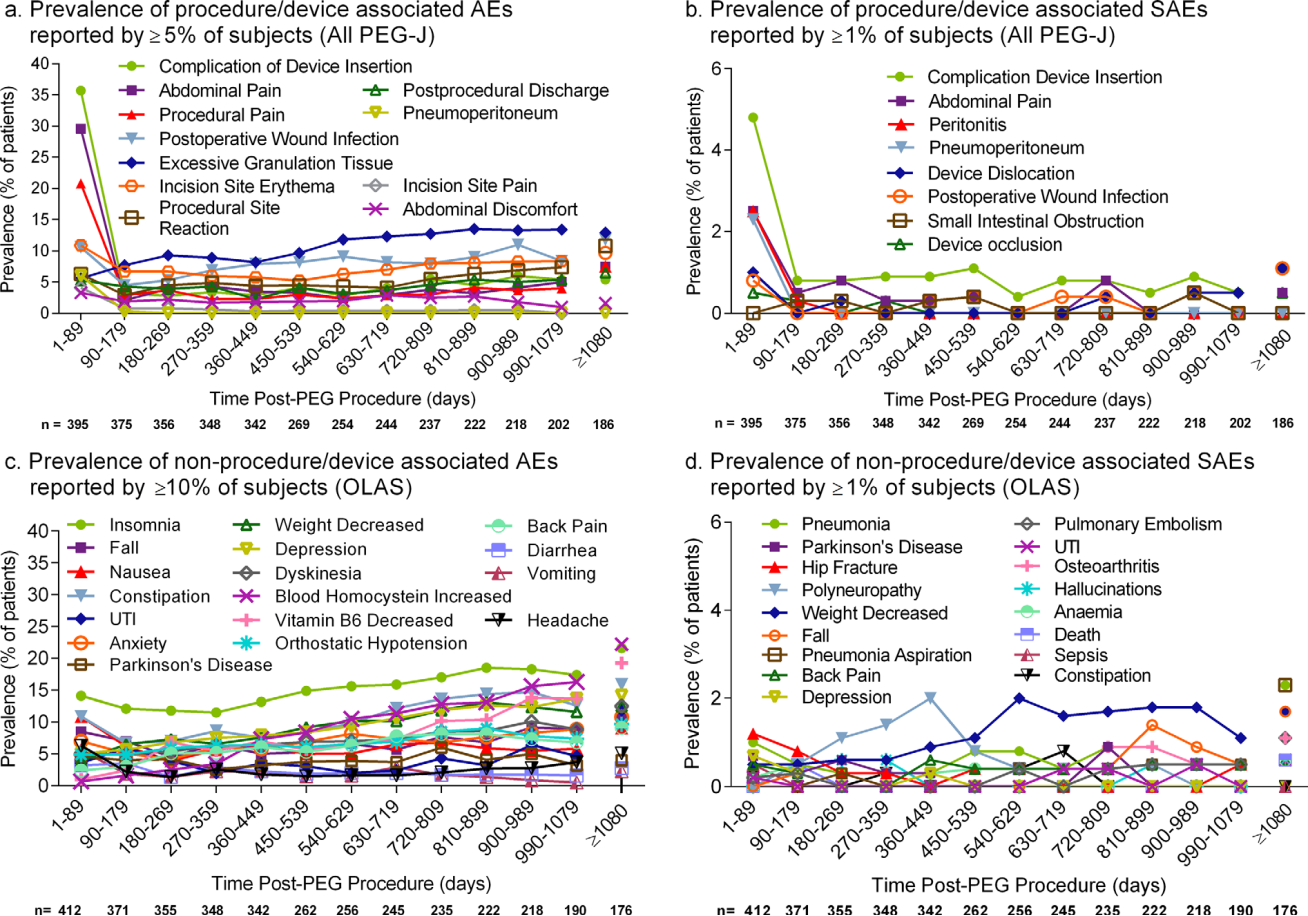
Prevalence of adverse events (AEs) and serious AEs (SAEs) over time. A single event could be coded to ≥1 preferred term. The AE term “Parkinson's disease” refers to the reemergence of Parkinson's symptoms, often because of interruption of drug delivery. All PEG‐J, data set of patients who had PEG‐J placement; OLAS, open‐label LCIG analysis data set; UTI, urinary tract infection.

At least 1 procedure/device AE occurred in 300 patients (76%); the most frequently reported are summarized in Table 2. The majority of AEs coded as a complication of device insertion were also coded to 1 or more of the following PTs: abdominal pain, abdominal discomfort, abdominal distension, flatulence, or pneumoperitoneum. Procedure/device serious AEs (SAEs) occurred in 68 patients (17%); the most frequently reported are summarized in Table 2. The prevalence of procedure/device AEs (Fig. 2a) and SAEs (Fig. 2b) was highest after the procedure, decreased substantially afterward and then stabilized over time. The majority of procedure/device AEs that occurred within the first 2 weeks resolved within the titration period (17% were “persistent”); see Table 2.

**Table 2:** Incidence of procedure/device‐associated adverse events (AEs) and serious AEs (SAEs), all PEG‐J, n = 395

	n (%)		n (%)
Any AE	300 (76)	Any SAE	68 (17)
Titration period	244 (62)	Titration period	33 (8.4)
Maintenance period^a^	230 (60)	Maintenance period^a^	42 (11)
Persistent	69 (17)	Persistent	8 (2.0)
AEs occurring in ≥ 5% of patients by PT		SAEs occurring in ≥ 1% of patients by PT	
Complication of device insertion^a^	160 (41)	Complication of device insertion^a^	33 (8.4)
Abdominal pain	142 (36)	Abdominal pain	17 (4.3)
Procedural pain	107 (27)	Peritonitis	11 (2.8)
Postoperative wound infection	104 (26)	Device dislocation	9 (2.3)
Incision site erythema	87 (22)	Pneumoperitoneum	9 (2.3)
Excessive granulation tissue	86 (22)	Postoperative wound infection	7 (1.8)
Procedural site reaction	65 (16)	Device occlusion	4 (1.0)
Postprocedural discharge	51 (13)	Small intestinal obstruction	4 (1.0)
Pneumoperitoneum	24 (6.1)		
Incision site pain	22 (5.6)		
Abdominal discomfort	20 (5.1)		

A single event could be coded to ≥1 preferred term. Titration period is onset during days 1 through 28; maintenance Period is onset on or after day 29; persistent is event onset during titration period and continued into maintenance period with duration ≥7 days. OLAS, open‐label LCIG analysis data set; All PEG‐J, data set of patients who had PEG‐J placement; PT, MedDRA preferred term.

n = 382 for maintenance period. Exposure during maintenance period was up to day 1276, including 180 patients with overall exposure to PEG‐J ≥540 days.

a Events with this term were most often additionally coded to abdominal pain, abdominal discomfort, abdominal distension, flatulence, pneumoperitoneum.

During the PEG‐J exposure period (median, 986 days; range, 1‐1972 days; Supplemental Table 1), 102 patients (26%) had at least 1 PEG tube replacement, and 222 (56%) had at least 1 J‐tube replacement (Supplemental Table 4). At the end of the first year, 92% of patients retained the original PEG tube (Supplemental Fig. 1a), with an overall mean (SD) of 0.1 (0.3) PEG tube replacements; 63% retained the original J‐tube (Supplemental Fig. 1b), with an overall mean (SD) of 0.6 (1.0) J‐tube replacements (n = 394 patients). At the end of the second year, 82% retained the original PEG tube, and 49% retained the original J‐tube. Patients had a mean (SD) of 0.3 (0.7) PEG replacements and 1.4 (1.8) J‐tube replacements over the entire treatment period. There were no consistent trends in baseline characteristics for those who had multiple tube replacements.

### Device Complaints Related to the Product Quality (All PEG‐J Data Set, n = 395)

At least 1 device “complaint” (related to the product quality, reported separately from AEs) was reported in 371 patients (94%). The most frequently reported device “complaints” are summarized in Supplemental Table 5. Sixty‐six percent (259 of 395) had complaints that required no action. The most common action taken for a device malfunction was pump replacement. Tube migration was the most common reason for device dislocation, the majority of which were either proximal or distal dislocations of the J‐tube.

### Non‐Procedure/Device‐Associated LCIG Safety (OLAS Data Set, n = 412)

Non‐procedure/device AEs occurred in 379 LCIG‐exposed patients (92%) in the open‐label studies, and the most frequently reported are summarized in Table 3. The prevalence of non‐procedure/device AEs was relatively stable over time (Fig. 2c). There were 39 patients (9.5%) who had “sleep attacks” (Supplemental Table 6).

**Table 3:** Incidence of non‐procedure/device‐associated adverse events (AEs) and serious AEs (SAEs), OLAS, n = 412

	n (%)	Possibly or probably^a^ treatment related, n (%)		n (%)	Possibly or probably^a^ treatment related, n (%)
Any AE	379 (92)	288 (70)	Any SAE	171 (42)	66 (16)
Titration period	266 (65)		Titration period	26 (6.3)	
Maintenance period^b^	337 (89)		Maintenance period^b^	160 (42)	
Persistent	165 (40)		Persistent	13 (3.2)	
AEs occurring in ≥ 10% of patients by PT			SAEs occurring in ≥ 1% of patients by PT		
Insomnia	96 (23)	32 (7.8)	Pneumonia	20 (4.9)	0
Fall	95 (23)	31 (7.5)	Fall	10 (2.4)	3 (0.7)
Constipation	84 (20)	43 (10)	Hip fracture	10 (2.4)	0
Nausea	84 (20)	47 (11)	Parkinson's disease^c^	10 (2.4)	8 (1.9)
Urinary tract infection	71 (17)	3 (0.7)	Weight decreased	10 (2.4)	8 (1.9)
Vitamin B_6_ decreased	65 (16)	44 (11)	Polyneuropathy	9 (2.2)	8 (1.9)
Anxiety	61 (15)	20 (4.9)	Pneumonia aspiration	8 (1.9)	1 (0.2)
Dyskinesia	60 (15)	54 (13)	Anemia	5 (1.2)	2 (0.5)
Parkinson's disease^c^	59 (14)	42 (10)	Constipation	5 (1.2)	2 (0.5)
Weight decreased	59 (14)	50 (12)	Death	5 (1.2)	0
Blood homocysteine increased	56 (14)	47 (11)	Hallucination	5 (1.2)	5 (1.2)
Depression	55 (13)	13 (3.2)	Pulmonary embolism	5 (1.2)	0
Back pain	46 (11)	1 (0.2)	Back Pain	4 (1.0)	0
Orthostatic hypotension	44 (11)	23 (5.6)	Depression	4 (1.0)	2 (0.5)
Diarrhea	43 (10)	12 (2.9)	Osteoarthritis	4 (1.0)	0
Vomiting	43 (10)	27 (6.6)	Sepsis	4 (1.0)	1 (0.2)
Headache	41 (10)	5 (1.2)	Urinary tract infection	4 (1.0)	0

A single event could be coded to ≥1 preferred term. Titration period is onset during days 1 through 28; maintenance period is onset on of after day 29; persistent is event onset during titration period and continued into maintenance period with duration ≥7 days. OLAS, open‐label LCIG analysis data set; All PEG‐J, data set of patients who had PEG‐J placement; PT, MedDRA preferred term.

a Study investigator rated.

b n = 382 for maintenance period. Exposure during maintenance period was up to day 1276, including 180 patients with overall exposure to PEG‐J ≥ 540 days.

c Refers to the reemergence of Parkinson's symptoms, often because of interruption of drug delivery.

Non‐procedure/device SAEs occurred in 171 patients (42%); the most frequently reported non‐procedure/device SAEs are summarized in Table 3. The prevalence of the most frequently reported non‐procedure/device SAEs (Fig. 2d) was relatively stable over the treatment period. The prevalence of reported insomnia, depression, “weight decreased,” and “increased blood homocysteine” rose slightly over time (Fig. 2d). The most frequently reported non‐procedure/device AEs that were classified by study investigators as serious (ie, SAEs) *and* “possibly” or “probably” treatment system related were the reemergence of PD symptoms (2%) often because of interrupted drug delivery, polyneuropathy (2%), and “weight decreased” (2%); see Table 3.

Polyneuropathy was reported in 24 patients (5.8%); see Supplemental Table 6. Separately, AEs were analyzed by a more inclusive list of PT terms related to polyneuropathy (eg, neuralgia, peripheral neuropathy). This resulted in 39 patients (9.5%), and 12 of these patients (2.9%) reported medical histories that included neuropathy or known risk factors for neuropathy. Patients with polyneuropathy were treated at the investigators' discretion. Vitamin levels were subsequently added to the study protocol to monitor for vitamin deficiencies that could be risk factors for polyneuropathy. As assessments of vitamins B_6_ and B_12_ and other laboratory tests associated with neuropathy were not required at baseline, the causality between LCIG, vitamin deficiencies, and polyneuropathy could not be determined.

“Weight decreased” was reported in 59 patients (14%); see Table 3. Furthermore, patients who had a weight loss of at least 7% from baseline to any point and continued to have an additional weight loss of at least 4.5 kg (10 lb) afterward were identified (n = 37, 9.0%) for additional review. The majority of these patients (24 of 37) were overweight (BMI ≥ 25 to ≤ 29.9 kg/m^2^, n = 14) or obese (BMI ≥ 30 kg/m^2^, n = 10) at baseline, and 3 had a low BMI ( < 18.5 kg/m^2^) at final. Any subsequent treatment for weight loss was provided at the investigator's discretion.

At baseline, patients were excluded if they had an impulse control disorder (ICD) that study investigators considered significant; however, there were 15 patients (3.6%) included in these studies who screened positive for an ICD in the MIDI at baseline, 5 of whom had a positive MIDI during the study. At the data cutoff, there were 26 patients (6.1%) who had at least 1 compulsive behavior reported in the MIDI: 6 (1.5%) with pathological gambling, 5 (1.2%) with compulsive buying, and 19 (4.6%) with compulsive sexual behavior. Within the “abuse liability” search strategy (MedDRA coding most relevant to ICD, see Patients and Methods section), there were 32 patients (7.8%) who had AEs related to an ICD; the most relevant AEs were impulsive behavior (1.9%), dopamine dysregulation syndrome (0.5%), ICD (0.5%), pathological gambling (0.5%), and compulsive shopping (0.2%). Any subsequent treatment of the patient for the ICD was at the discretion of the investigator. For the patients with events of impulse control who were receiving extra LCIG doses, adjusting the LCIG treatment regimen typically led to resolution of the initial event.

### Premature Discontinuations Because of Adverse Events

Overall, 17% of patients (72 of 412 in OLAS) discontinued LCIG treatment because of an AE (either associated with the procedure/device or not); the most frequently reported AEs that led to discontinuation by 3 or more patients were complication of device insertion (2.4%), death (1.2%), abdominal pain (1.0%), pneumonia (1.0%), myocardial infarction (0.7%), and fall (0.7%) (OLAS). The proportion who discontinued because of a procedure/device AE was 4.8% (19 of 395 in All PEG‐J), with discontinuation of 2.0% of patients during the 28‐day titration phase and 2.8% during the maintenance phase.

### Deaths

There were 34 deaths (8.3%) in the phase 3 studies (n = 412), with 32 considered by investigators to be “unrelated” or “unlikely related” to the treatment system and 2 considered to be “possibly related.” Two of the unrelated deaths (0.5%) were a result of suicide on days 317 and 370, and both patients had a relevant history of depression. One patient (0.2%) whose death was considered “possibly related” had a cardiac arrest (SAE) on day 491 of treatment. The other had “intestinal dilatation” (SAE) on day 1071 with additional findings of pneumotosis intestinalis, portal venous gas, intestinal air fluid levels, markedly distended stomach, and air leakage from the feeding tube during coughs.

## Discussion

This is the largest prospective study to date evaluating safety of LCIG. The safety of LCIG was evaluated in 412 patients, and as of March 31, 2014, included more than 950 patient‐years of data, with a mean exposure of 911 days in a population of patients with advanced PD.^17^ However, as this was an analysis of data integrated from 4 studies, there are several limitations to consider from the outset. Selection bias may have influenced patient inclusion. More rigorous follow‐up evaluations may have reduced the overall risk of SAEs, even though this methodology might also inflate the frequency of all reported AEs compared to routine practice. Because the study‐required method of safety reporting does not always translate to clinical practice, safety from a recent large observational report may be closer to what is seen in routine care.^17^ In the present analysis, safety data from 1 double‐blind and 3 open‐label studies were included. Even though open‐label studies have multiple limitations, the majority of safety data for new therapies are often derived from open‐label studies.^14^ Although there may be regional variation within clinical practice,^18^ GI proceduralists were given standardized procedure/device training prior to participating in the study to limit the impact on treatment and AE reporting. Postprocedural data collection was also tightly monitored to ensure an accurate assessment of the safety of LCIG treatment. Within the context of these limitations, meaningful conclusions can still be derived for both the procedure/device and the non‐procedure/device safety of LCIG. These data represent important considerations when evaluating the appropriateness of treatment with LCIG.

A substantial number of patients experienced non‐procedure/device SAEs; however, the events with the highest incidence were consistent with AEs frequently reported in an older patient population (pneumonia), related to the underlying disease (fall and hip fracture),^19^, ^20^, ^21^, ^22^ or known to be associated with dopaminergic therapy (insomnia, nausea, and hallucination).^23^, ^24^, ^25^, ^26^ The incidence of ICDs and “sleep attacks” was also consistent with other dopaminergic treatments, including oral levodopa.^27^, ^28^, ^29^ Although 2% of patients had SAEs of polyneuropathy, meaningful conclusions about the root cause could not be made without baseline values for vitamins B_6_ and B_12_ or systematic electrodiagnostic evaluations, which were lacking in these studies. Considering the non‐procedure/device events alone, the safety profile of LCIG was comparable to the established safety profile of the oral formulation of levodopa‐carbidopa.^30^, ^31^

Previously, the safety of the PEG‐J placement procedure in advanced PD patients was relatively unknown. However, the majority of procedure/device AEs reported by patients who had a PEG‐J placement in these studies were consistent, in nature and incidence, with medically recognized complications of the procedure in non‐PD patient populations.^32^, ^33^, ^34^, ^35^, ^36^ Although rare, some procedure/device AEs can be life threatening, including intestinal perforation, peritonitis, and intestinal hemorrhage. However, most procedure/device AEs resolved within the first 28 days of treatment. Despite the level of mobility of this patient population being unlike a typical chair/bedbound population of patients with a PEG‐J for other indications,^34^, ^35^ the PEG and J‐tubes proved durable. An independent GI adjudication committee reviewed the procedure/device‐associated safety data and determined that rates were consistent with the literature, and no specific events limited its use in this patient population.^16^

The rate of discontinuation because of an AE (either associated or not associated with a procedure/device) was 17% and remained stable after the titration period. This rate was slightly higher than shorter studies for oral dopaminergic treatments, which ranged from 10% to 13%^23^, ^24^, ^25^, ^26^ and comparable to other studies for LCIG, which ranged from 15% to 22%.^37^, ^38^, ^39^ Considering the length of exposure, the percentage of patients who discontinued LCIG because of an AE supports its overall tolerability, but may also reflect the influence of committing to having a PEG‐J. One death of a patient with “intestinal dilatation” was probably related to the mode of medication delivery. The majority of deaths were consistent with the mortality profile in this patient population,^40^, ^41^, ^42^ including suicide.^40^, ^43^ Nonetheless, closely monitoring depressive states and suicidal ideation is recommended.

In conclusion, we have presented the largest, longest‐term safety data set from prospective clinical studies for LCIG to date. Given that conclusions regarding polyneuropathy, weight loss, and psychiatric symptoms were limited, further systematic surveillances of these are warranted. Within the limitations of this analysis, procedure/device AEs were common, expected for the known risks associated with a PEG‐J, and the most common cause of discontinuation. Non‐procedure/device AEs were consistent with what would be expected for the patient population and extent of dopaminergic exposure. This study suggests that despite the high incidence of patients with AEs, LCIG can be used in a safe and tolerable manner for the treatment of motor fluctuations that are inadequately controlled by other PD medications in levodopa‐responsive patients with advanced PD.

## Author Roles

Research project — Conception, K.C., J.A.B.; Organization, K.C., J.A.B.; Execution, A.E.L., R.L.R., J.T.B., S.C., C.Z., A.J.E., J.T.S., H.H.F., M.F.L., P.O., D.A.S., V.S.C.F., F.K., P.D., N.S., K.C., J.A.B., J.D. Statistical analysis — Design, W.Z. R.; Execution, W.Z. R.; Review and critique, W.Z.R., A.E.L., A.F., S.E., K.C., J.A.B., J.D. Manuscript —Writing of the first draft, A.E.L., A.F., J.D.; Review and critique, A.E.L., R.L.R., J.T.B., S.C., C.Z., A.J.E., J.T.S., H.H.F., M.F.L., P.O., D.A.S., V.S.C.F., A.F., F.K., P.D., N.S., W.Z.R.., S.E., K.C., J.A.B., J.D.

## Full financial disclosures of all authors for the past year

A.E.L. has served as an adviser for Abbvie Inc., Allon Therapeutics, Avanir Pharmaceuticals, Biogen Idec, Boerhinger‐Ingelheim, Ceregene, Lilly, Medtronic, Merck, Novartis, NeuroPhage Pharmaceuticals, Teva, and UCB; received honoraria from Teva, UCB, AbbVie Inc.; received grants from Brain Canada, Canadian Institutes of Health Research, Edmond J Safra Philanthropic Foundation, Michael J. Fox Foundation, the Ontario Brain Institute, National Parkinson Foundation, Parkinson Society Canada, Tourette Syndrome Association, W. Garfield Weston Foundation; received publishing royalties from Saunders, Wiley‐Blackwell, Johns Hopkins Press, and Cambridge University Press; and has served as an expert witness in cases related to the welding industry. R.L.R. has received research support from Abbvie Inc., Allergan, Auspex, Biotie Therapeutics, Dystonia Coalition, Huntington Study Group, Ipsen, Merz Pharmaceuticals, National Parkinson Foundation, NIH/NINDS, Parkinson's Study Group, Chelsea, and Auspex, but has no owner interest in any pharmaceutical company; and honoraria from PeerView Institute for Medical Education, Merz, Lundbeck, Chelsea, Abbvie Inc., and the CME Meeting. J.T.B. served as a consultant and/or scientific adviser for AbbVie Inc., Auspex, Lundbeck, and Oakstone Medical Publishing; and received research support from Michael J. Fox Foundation, NIH/NINDS, Auspex, and Abbvie Inc. S.C. has received research support from AbbVie Inc. and Allergan, and served as an consultant, adviser and/or speaker for Teva, AbbVie, Novartis, Merz, Allergan, and UCB. C.Z. has served as an adviser for Abbvie Inc. and Merz and has received honoraria from Teva, UCB, US World Meds, Lundbeck, and Merz. A.J.E. has been supported by the K23 career development award (NIMH, 1K23MH092735); received research support from CleveMed/Great Lakes Neurotechnologies, and the Michael J Fox Foundation; personal compensation as a consultant and/or adviser for Abbvie Inc., Chelsea Therapeutics, Teva, Impax, Merz, Pfizer, Acadia, Solstice Neurosciences, Eli Lilly, and USWorldMeds; royalties from Lippincott Williams & Wilkins and Cambridge University Press; and honoraria from UCB, Teva, the American Academy of Neurology, and the Movement Disorders Society. A.J.E has served as Associate Editor of *Movement Disorders, Frontiers in Movement Disorders*, and *Journal of Clinical Movement Disorders* and on the editorial boards of *Parkinsonism and Related Disorders* and *The European Neurological Journal*. J.T.S. has served as an adviser for AbbVie Inc., a speaker for Teva, and has received research support from AbbVie Inc., Biotie, NIH and Veterans Administration. H.H.F. has received research support from Abbvie Inc., Acadia, Biotie Therapeutics, EMD‐Serono, Merck, Michael J. Fox Foundation, Movement Disorders Society, NIH/NINDS, Novartis, Parkinson Study Group, Synosia, Teva, but has no owner interest in any pharmaceutical company; honoraria from Prime Education Inc, Ohio State University, International Parkinson and Movement Disorders Society, Carling Communications, as a speaker in CME events; honoraria from Auspex Pharmaceuticals, Britannia, GE Health Care, Lundbeck, Merz Pharmaceuticals, and Pfizer Pharmaceuticals, as a consultant; a stipend from International Parkinson and Movement Disorders Society for serving as Medical Editor of the MDS website; royalties from Demos Publishing for serving as a book author/editor; and has contracts with Abbvie Inc. and Merz Pharmaceuticals for his role as a member of the Global Steering Committee for LCIG studies. H.H.F. serves as the Head Principal Investigator for the Xeomin Registry Study, the Chair of the Publication Committee for Xeomin Studies (Merz Pharmaceuticals); and a member of the Publication Committee for Dysport studies (Ipsen Pharmaceuticals) but he does not receive any personal compensation for these roles. M.F.L. has served as a consultant, adviser, and/or lecturer for Teva, US World Meds, Merz, UCB, AbbVie Inc., Acadia, Auspex, Lundbeck, and Baxter and has received research support from NIH, AbbVie Inc., Parksinson's Study Group, US World Meds, Michael J. Fox Foundation, Synosia Pharmaceuticals, Merz, Ipsen, Pharma two B, Civitas, and Chelsea Therapeutics. P.O. has served as consultant and/or lecturer for AbbVie Inc., Britannia, Ipsen, Lundbeck, Nordic Infucare and UCB, and has received honoraria from the Movement Disorder Society. D.A.S. was a medical monitor for the studies through a contract with his employer, Quintiles, and AbbVie Inc. V.S.C.F. receives a salary from NSW Health, has received research support from the National Health and Medical Research Council of Australia, and is on Advisory Boards and/or has received travel grants from Abbvie Inc., Allergan, Boehringer‐Ingelheim, Hospira, Ipsen, Lundbeck, Novartis, Parkinson's KinetiGraph, Solvay and UCB. A.F. has served as an adviser for Abbvie Inc.; consultant for UCB pharma, Medtronic, Boston Scientific, and Abbvie Inc; and received research support from University of Toronto and Michael J. Fox Foundation; and honoraria from UCB, Medtronic, Boston Scientific, Abbvie Inc. and Teva for serving as speaker. F.K. has served as an adviser for UCB and Archimedes; lecturer for AbbVie Inc., Ipsen and Lundbeck; and received research support from the German Research Foundation. P.V.D. has served as a consultant for AbbVie Inc., Boston Scientific, Olympus American, and Cook Medical. N.S. has served as a consultant, scientific adviser and lecturer for AbbVie Inc. J.D., a former employee of AbbVie Inc, is employed by Cynapsus Therapeutics. W.Z. R., S.E., K.C., and J.A.B. are employees of AbbVie Inc. and hold stock or stock options.

## Supporting information

Additional Supporting Information may be found in the online version of this article at the publisher's web‐site.

Supplementary Information Figures.

Supplementary Information Table 1.

Supplementary Information Table 2.

Supplementary Information Table 3.

Supplementary Information Table 4.

Supplementary Information Table 5.

Supplementary Information Table 6.
