# Supplementary material for: Integrated safety of levodopa‐carbidopa intestinal gel from prospective clinical trials
Source: Mov Disord. 2015 Dec 23;31(4):538–46. doi: 10.1002/mds.26485 (PMC5064722; doi:10.1002/mds.26485)
Supplement: Supplementary file 1 — Supplementary Information Figures. [file MDS-31-538-s001.doc]

**Supplemental Figure Legends**

**
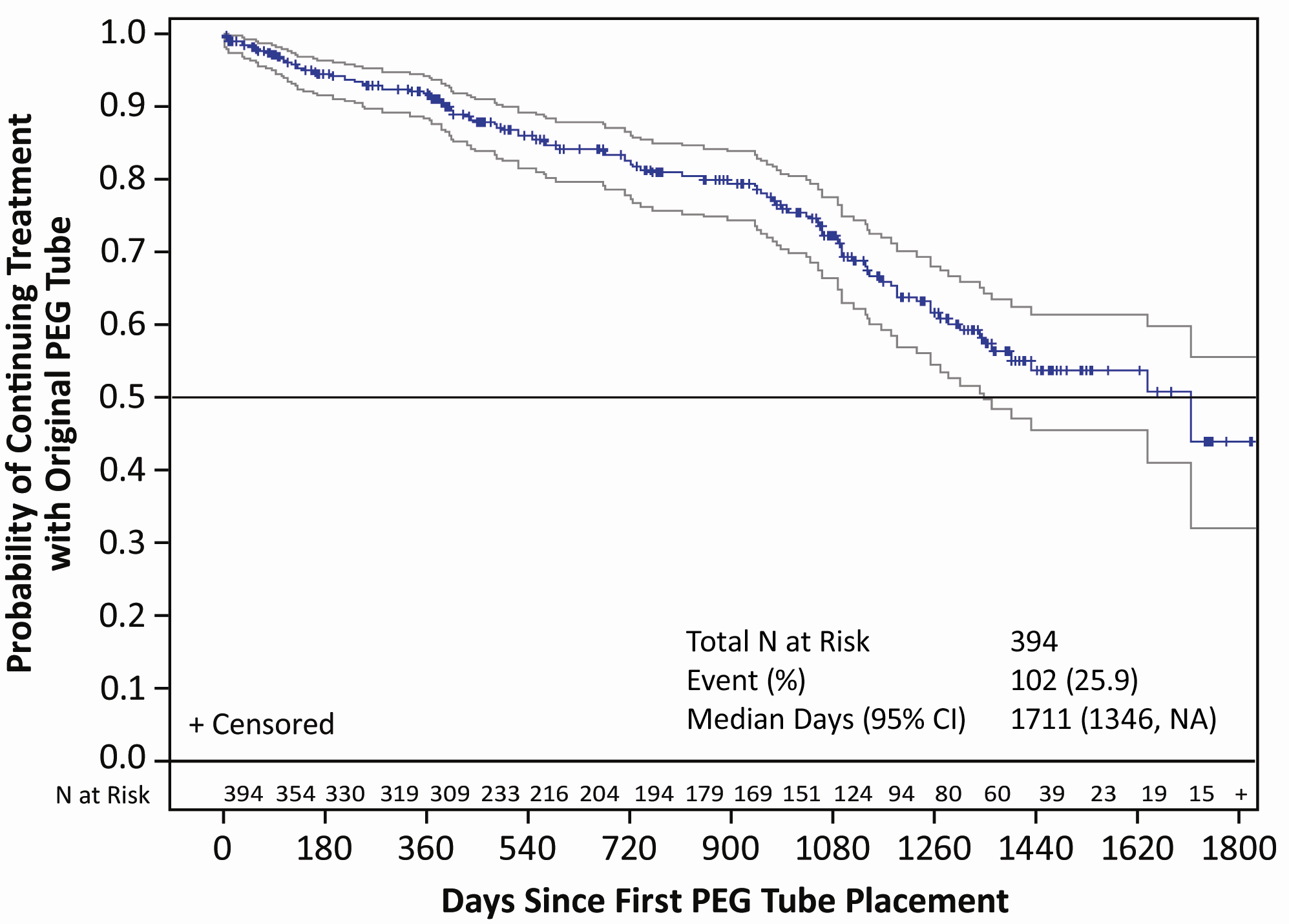

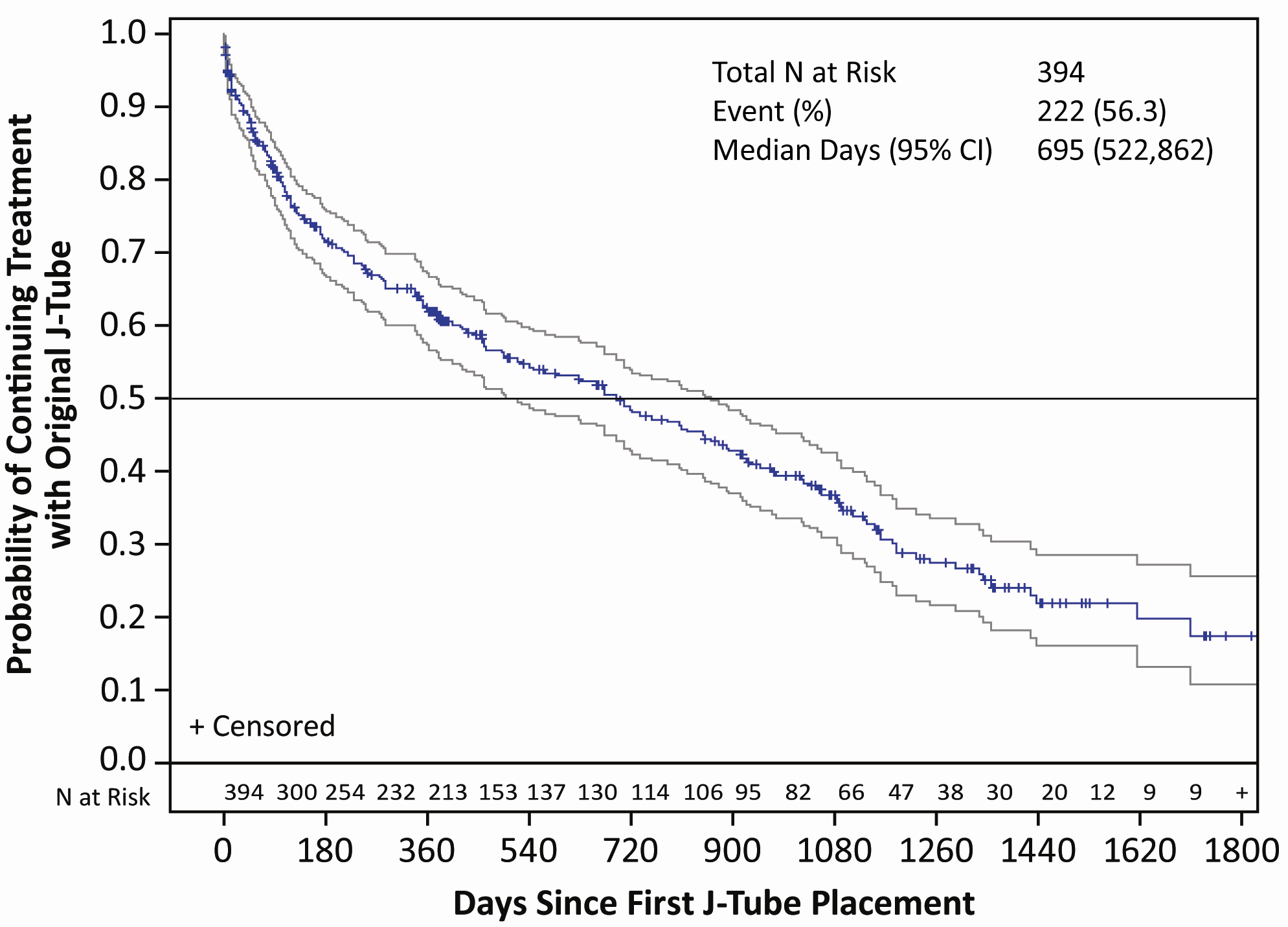
**

**Supplemental Figure 1. Time to First Tube Replacement (All PEG-J, N=395)**

All PEG-J = dataset of patients who had PEG-J placement. PEG-J = percutaneous endoscopic gastrojejunostomy; J = jejunal

**Supplemental Table Legends**

**Supplemental Table 1. Total Exposure to LCIG and PEG-J**

PEG = percutaneous endoscopic gastrostomy; J = jejunal; LCIG = levodopa-carbidopa intestinal gel; All PEG-J = dataset of patients who had PEG-J placement; OLAS = open-label LCIG analysis dataset

**Supplemental Table 2. Summary Table of LCIG Subgroup Analyses by Patient Incidence of Procedure/Device Associated Adverse Events (All PEG-J, N=395)**

All PEG-J = dataset of patients who had PEG-J placement; LCIG = levodopa-carbidopa intestinal gel; IOE = Israel, Oceania and Western European countries, ACE = Asian and Central European countries

**Supplemental Table 3. Summary Table of LCIG Subgroup Analyses by Patient Incidence of Non-Procedure/Device Adverse Events (OLAS, N=412)**

OLAS = open-label LCIG analysis dataset; LCIG = levodopa-carbidopa intestinal gel; IOE = Israel, Oceania and Western European countries, ACE = Asian and Central European countries

**Supplemental Table 4**. **Number of PEG and J-Tube Replacements (All PEG-J, N=395)**

PEG = percutaneous endoscopic gastrostomy; J = jejunal; All PEG-J = dataset of patients who had PEG-J placement

**Supplemental Table 5**. **Device Complaints Reported by ≥5% of Patients (All PEG-J, N=395)**

All PEG-J = dataset of patients who had PEG-J placement

**Supplemental Table 6. Incidence (≥5 and <10%) of Non-Procedure/Device Associated Adverse Events (AEs) (OLAS, N=412)**

A single event could be coded to ≥1 preferred term. OLAS = open-label LCIG analysis dataset; PT = MedDRA preferred term
