# Supplementary material for: Integrated safety of levodopa‐carbidopa intestinal gel from prospective clinical trials
Source: Mov Disord. 2015 Dec 23;31(4):538–46. doi: 10.1002/mds.26485 (PMC5064722; doi:10.1002/mds.26485)
Supplement: Supplementary file 2 — Supplementary Information Table 1. [file MDS-31-538-s002.docx]

**Supplemental Table 1**: Total Exposure to PEG-J and LCIG

| **Exposure to:** | **PEG-J** | **LCIG** |
| --- | --- | --- |
| **Duration Interval, days** | **All PEG-J (N=395)**  **n (%)** | **OLAS (N=412)**  **n (%)** |
| ≥6 months (180 days) | 351 (89) | 350 (85) |
| ≥1 year (365 days) | 337 (85) | 336 (82) |
| ≥18 months (540 days) | 252 (64) | 252 (61) |
| ≥2 years (730 days) | 235 (59) | 233 (57) |
| ≥30 months (900 days) | 214 (54) | 210 (51) |
| ≥3 years (1095 days) | 165 (42) | 159 (39) |
| ≥42 months (1260 days) | 130 (33) | 124 (30) |
| **Mean ± SD, days** | 904 ± 546 | 854 ± 564 |
| **Median (Range), days** | 986 (1 – 1972) | 911 (1 – 1980) |
| **Total exposure, patient-years** | 978 | 963 |

PEG = percutaneous endoscopic gastrostomy; J = jejunal; LCIG = levodopa-carbidopa intestinal gel; All PEG-J = dataset of patients who had PEG-J placement; OLAS = open-label LCIG analysis dataset
