# Supplementary material for: Integrated safety of levodopa‐carbidopa intestinal gel from prospective clinical trials
Source: Mov Disord. 2015 Dec 23;31(4):538–46. doi: 10.1002/mds.26485 (PMC5064722; doi:10.1002/mds.26485)
Supplement: Supplementary file 3 — Supplementary Information Table 2. [file MDS-31-538-s003.docx]

**Supplemental Table 2.** Summary Table of LCIG Subgroup Analyses by Patient Incidence of Procedure/Device Adverse Events (All PEG-J, N=395)

| **Subgroup** | **N** | **Any AE,**  **n (%)** | **Any SAE,**  **n (%)** | **Discontinued due to AE,**  **n (%)** |
| --- | --- | --- | --- | --- |
| Age^a^, < 65 years | 192 | 147 (77) | 27 (14) | 8 (4.2) |
| ≥ 65 years | 203 | 150 (74) | 35 (17) | 10 (4.9) |
| Gender, female | 164 | 127 (77) | 32 (20) | 11 (6.7) |
| male | 231 | 170 (74) | 30 (13) | 7 (3.0) |
| Race, white | 367 | 270 (74) | 57 (16) | 13 (3.5) |
| Asian | 24 | 23 (96) | 3 (13) | 5 (21) |
| other | 4 | 4 (100) | 2 (50) | 0 |
| BMI^a^, < 25 kg/m^2^ | 216 | 167 (77) | 34 (16) | 11 (5.1) |
| ≥ 25 kg/m^2^ | 174 | 125 (72) | 27 (16) | 7 (4.0) |
| Duration of PD ^a^, < 10 years | 157 | 125 (80) | 20 (13) | 9 (5.7) |
| ≥ 10 years | 238 | 172 (72) | 42 (18) | 9 (3.8) |
| Region, North America | 142 | 132 (93) | 29 (20) | 6 (4.2) |
| IOE | 166 | 117 (70) | 21 (13) | 4 (2.4) |
| ACE | 87 | 48 (55)^b^ | 12 (14) | 8 (9.2) |

All PEG-J = dataset of patients who had PEG-J placement; LCIG = levodopa-carbidopa intestinal gel; IOE = Israel, Oceania and Western European countries, ACE = Asian and Central European countries

1. At baseline
2. There was a higher incidence of AEs in North America versus other regions; however this trend was not observed for SAEs or discontinuations due to AEs.
