# Supplementary material for: Integrated safety of levodopa‐carbidopa intestinal gel from prospective clinical trials
Source: Mov Disord. 2015 Dec 23;31(4):538–46. doi: 10.1002/mds.26485 (PMC5064722; doi:10.1002/mds.26485)
Supplement: Supplementary file 4 — Supplementary Information Table 3. [file MDS-31-538-s004.docx]

**Supplemental Table 3.** Summary Table of LCIG Subgroup Analyses by Patient Incidence of Non-Procedure/Device Adverse Events (OLAS, N=412)

| **Subgroup** | **N** | **Any AE,**  **n (%)** | **Any SAE,**  **n (%)** | **Discontinued due to AE,**  **n (%)** |
| --- | --- | --- | --- | --- |
| Age^a^, < 65 years | 201 | 183 (91) | 76 (38) | 26 (13) |
| ≥ 65 years | 211 | 200 (95) | 95 (45) | 32 (15) |
| Gender, female | 169 | 160 (95) | 76 (45) | 26 (15) |
| male | 243 | 223 (92) | 95 (39) | 32 (13) |
| Race, white | 381 | 353 (93) | 157 (41) | 48 (13) |
| Asian | 26 | 26 (100) | 11 (42) | 9 (35) |
| other | 5 | 4 (80) | 3 (60) | 1 (10) |
| BMI^a^, < 25 kg/m^2^ | 229 | 211 (92) | 90 (39) | 33 (14) |
| ≥ 25 kg/m^2^ | 178 | 167 (94) | 78 (44) | 25 (14) |
| Duration of PD ^a^, < 10 years | 159 | 151 (95) | 64 (40) | 30 (19) |
| ≥ 10 years | 253 | 232 (92) | 107 (42) | 28 (11) |
| Dopamine agonist, user | 84 | 83 (99) | 35 (42) | 12 (14) |
| non-user | 328 | 300 (92) | 136 (42) | 46 (14) |
| Region, North America | 143 | 142 (99) | 62 (43) | 17 (12) |
| IOE | 173 | 161 (93) | 77 (45) | 22 (13) |
| ACE | 96 | 80 (83) | 32 (33) | 19 (20) |

OLAS = open-label LCIG analysis dataset; LCIG = levodopa-carbidopa intestinal gel; IOE = Israel, Oceania and Western European countries, ACE = Asian and Central European countries

1. At baseline
