# Supplementary material for: Integrated safety of levodopa‐carbidopa intestinal gel from prospective clinical trials
Source: Mov Disord. 2015 Dec 23;31(4):538–46. doi: 10.1002/mds.26485 (PMC5064722; doi:10.1002/mds.26485)
Supplement: Supplementary file 5 — Supplementary Information Table 4. [file MDS-31-538-s005.docx]

**Supplemental Table 4**: Number of PEG and J-Tube Replacements (All PEG-J, N=395)

| **Tube Replacements** | **PEG tube, N (%)** | | **J-tube**  **N (%)** | |
| --- | --- | --- | --- | --- |
| **0** | 292 (74) | 172 (44) | |  |
| **1** | 78 (20) | 93 (24) | |  |
| **2** | 16 (4.1) | 47 (12) | |  |
| **3** | 5 (1.3) | 38 (9.6) | |  |
| **4** | 3 (0.8) | 20 (5.1) | |  |
| **5** | 0 | | 11 (2.8) | |
| **6** | 0 | 5 (1.3) | |  |
| **7** | 0 | 4 (1.0) | |  |
| **8** | 0 | 1 (0.3) | |  |
| **9** | 0 | 0 | |  |
| **10** | 0 | 2 (0.5) | |  |
| **11** | 0 | 0 | |  |
| **12** | 0 | 1 (0.3) | |  |

PEG = percutaneous endoscopic gastrostomy; J = jejunal; All PEG-J = dataset of patients who had PEG-J placement
