# Supplementary material for: Integrated safety of levodopa‐carbidopa intestinal gel from prospective clinical trials
Source: Mov Disord. 2015 Dec 23;31(4):538–46. doi: 10.1002/mds.26485 (PMC5064722; doi:10.1002/mds.26485)
Supplement: Supplementary file 6 — Supplementary Information Table 5. [file MDS-31-538-s006.docx]

**Supplemental Table 5**: Device Complaints Reported by ≥5% of Patients (All PEG-J, N=395)

|  | **N (%)** |
| --- | --- |
| **Any Device Complaint^a^** | 371 (94) |
| Device malfunction^b^ | 233 (59) |
| Complication of device insertion | 207 (52) |
| Device dislocation | 185 (47) |
| Medical device site reaction | 173 (44) |
| Device occlusion | 151 (38) |
| Device related infection | 115 (29) |
| Device connection issue | 108 (27) |
| Device breakage | 70 (18) |
| Device leakage | 65 (16) |
| Unintentional medical device removal by patient | 62 (16) |
| Procedural Pain | 24 (6.1) |

All PEG-J = dataset of patients who had PEG-J placement

1. A single device issue could be coded to >1 compliant term. Device complaints include any malfunction or deterioration in the characteristics and/or performance of a device. These may or may not have been reported in association with an AE or SAE.
2. Device malfunctions include pump complaints.
