# Supplementary material for: Integrated safety of levodopa‐carbidopa intestinal gel from prospective clinical trials
Source: Mov Disord. 2015 Dec 23;31(4):538–46. doi: 10.1002/mds.26485 (PMC5064722; doi:10.1002/mds.26485)
Supplement: Supplementary file 7 — Supplementary Information Table 6. [file MDS-31-538-s007.docx]

**Supplemental Table 6**. Incidence (≥5 and <10%) of Non-Procedure/Device Associated Adverse Events (AEs) (OLAS, N=412)

|  | **N (%)** | | **Possibly or Probably^a^ Treatment Related, N (%)** |
| --- | --- | --- | --- |
| **Any AE** | | 379 (92) | 288 (70) |
| **AEs Occurring in ≥5% and <10% of Patients by PT** | | |  |
| Sleep attacks | | 39 (9.5) | 23 (5.6) |
| Arthralgia | | 33 (8.0) | 1 (0.2) |
| Dyspepsia | | 33 (8.0) | 16 (3.9) |
| Hallucination | | 31 (7.5) | 27 (6.6) |
| Pain in extremity | | 30 (7.3) | 2 (0.5) |
| Dizziness | | 28 (6.8) | 11 (2.7) |
| Musculoskeletal pain | | 27 (6.6) | 6 (1.5) |
| Oropharyngeal pain | | 27 (6.6) | 17 (4.1) |
| Pneumonia | | 27 (6.6) | 1 (0.2) |
| Decreased appetite | | 26 (6.3) | 17 (4.1) |
| Vitamin B_6_ deficiency | | 26 (6.3) | 23 (5.6) |
| Fatigue | | 25 (6.1) | 7 (1.7) |
| Laceration | | 24 (5.8) | 0 |
| Muscle spasms | | 24 (5.8) | 7 (1.7) |
| Polyneuropathy | | 24 (5.8) | 20 (4.9) |
| Anemia | | 22 (5.3) | 7 (1.7) |
| Dyspnoea | | 22 (5.3) | 7 (1.7) |
| Pain | | 22 (5.3) | 6 (1.5) |
| Basal cell carcinoma | | 21 (5.1) | 2 (0.5) |
| Dystonia | | 21 (5.1) | 10 (2.4) |

A single event could be coded to ≥1 preferred term. OLAS = open-label LCIG analysis dataset; PT = MedDRA preferred term

a. Study investigator-rated
